# Hepatocyte cholesterol content modulates glucagon receptor signalling

**DOI:** 10.1016/j.molmet.2022.101530

**Published:** 2022-06-16

**Authors:** Emma Rose McGlone, T. Bertie Ansell, Cecilia Dunsterville, Wanling Song, David Carling, Alejandra Tomas, Stephen R. Bloom, Mark S.P. Sansom, Tricia Tan, Ben Jones

**Affiliations:** 1 Department of Metabolism, Digestion and Reproduction, Imperial College London, London W12 0NN, United Kingdom; 2 Department of Surgery and Cancer, Imperial College London, London W12 0NN, United Kingdom; 3 Department of Biochemistry, University of Oxford, Oxford OX1 3QU, United Kingdom; 4 Cellular Stress Research Group, MRC London Institute of Medical Sciences, Imperial College London, London W12 0NN, United Kingdom

**Keywords:** Glucagon, Glucagon receptor, Cholesterol, Cell membrane, Non-alcoholic fatty liver disease, Type 2 diabetes mellitus, GCGR, glucagon receptor, T2D, type 2 diabetes mellitus, NAFLD, non-alcoholic fatty liver disease, GPCR, G protein-coupled receptor, GLP-1R, glucagon-like peptide 1 receptor, MβCD, methyl-β-cyclodextrin, MD, molecular dynamics

## Abstract

**Objective:**

To determine whether glucagon receptor (GCGR) actions are modulated by cellular cholesterol levels.

**Methods:**

We determined the effects of experimental cholesterol depletion and loading on glucagon-mediated cAMP production, ligand internalisation and glucose production in human hepatoma cells, mouse and human hepatocytes. GCGR interactions with lipid bilayers were explored using coarse-grained molecular dynamic simulations. Glucagon responsiveness was measured in mice fed a high cholesterol diet with or without simvastatin to modulate hepatocyte cholesterol content.

**Results:**

GCGR cAMP signalling was reduced by higher cholesterol levels across different cellular models. *Ex vivo* glucagon-induced glucose output from mouse hepatocytes was enhanced by simvastatin treatment. Mice fed a high cholesterol diet had increased hepatic cholesterol and a blunted hyperglycaemic response to glucagon, both of which were partially reversed by simvastatin. Simulations identified likely membrane-exposed cholesterol binding sites on the GCGR, including a site where cholesterol is a putative negative allosteric modulator.

**Conclusions:**

Our results indicate that cellular cholesterol content influences glucagon sensitivity and indicate a potential molecular basis for this phenomenon. This could be relevant to the pathogenesis of non-alcoholic fatty liver disease, which is associated with both hepatic cholesterol accumulation and glucagon resistance.

## Highlights

• Hepatic cholesterol accumulation induces glucagon resistance.
• Cholesterol binding sites on the glucagon receptor differ in the active state.
• Increased membrane cholesterol may directly induce glucagon resistance in NAFLD.

## 1 Introduction

Glucagon is a key regulator of hepatic metabolism: as well as increasing glucose production to counteract hypoglycaemia, it reduces liver fat by decreasing *de novo* lipogenesis and increasing fatty acid oxidation [1,2]. Individuals with type 2 diabetes mellitus (T2D) and/or non-alcoholic fatty liver disease (NAFLD) exhibit hepatic glucagon resistance [3,4], which in turn contributes to worsening of steatosis by blocking the beneficial effects of glucagon on hepatic fat. To date, the mechanism behind this phenomenon is incompletely understood [5]. Hepatic cholesterol accumulation is a feature of NAFLD and, interestingly, the degree of both glucagon resistance [6] and hepatic cholesterol accumulation [7,8] are correlated with histological severity of the disease.

The glucagon receptor (GCGR) is a prototypical class B G protein-coupled receptor (GPCR). It is increasingly appreciated that the functions of cell surface GPCRs are heavily modulated by other membrane components [9], including lipids [10]. This can occur either through direct lipid–receptor interactions [11], or indirectly, e.g. by altering the properties of the membrane bilayer [12]. Cholesterol is known to alter the conformation of some GPCRs by directly binding to allosteric sites [13,14]; it is also a key structural component of the plasma membrane that controls the distribution and spatial coupling between surface receptors and intracellular mediators [15]. We have recently demonstrated that cholesterol depletion affects signalling and endomembrane trafficking of the closely related glucagon-like peptide 1 receptor (GLP-1R) [16]. To date, the relevance of cellular cholesterol to GCGR signalling has not been explored experimentally.

The aim of this study was to investigate whether hepatocyte cholesterol content affects GCGR signalling and its downstream physiological correlates. We demonstrate that enrichment of cholesterol *in vitro* and *in vivo* decreases glucagon responsiveness, whereas cholesterol depletion has the opposite effect. Molecular dynamics (MD) simulations identify likely cholesterol binding sites on the GCGR which may allosterically regulate its function. Our results indicate that hepatocyte cholesterol content influences hepatic glucagon sensitivity and pinpoint a potential molecular basis for this phenomenon.

## 2 Materials and methods

### 2.1 Cell culture and primary hepatocyte isolation

Huh7-GCGR cells [17] were cultured at 37 °C in 5% CO_2_ in DMEM supplemented with 10% FBS and 1% penicillin/streptomycin, with 1% G418 for the latter (Thermo Fisher). Hepatocytes from male adult C57Bl/6J mice were isolated using collagenase perfusion, as previously described [18], and were either assayed in suspension or plated and cultured at 37 °C, 5% CO_2_ in M199 supplemented with 1% penicillin/streptomycin, 1% BSA, 2% Ultroser G, 100 nM T3, 100 nM dexamethasone and 100 nM insulin (Thermo Fisher). After 5 h, medium was changed to M199 with 1% penicillin/streptomycin, 100 nM dexamethasone and 10 nM insulin for serum starvation. Primary hepatocytes from human cadaveric donors were obtained from Biopredic International (St Gregoire, France) and assayed in suspension. Donor characteristics are listed in Supplementary Table 1.

### 2.2 Peptides

Glucagon and a fluorescent glucagon analogue, “FITC-GCG” [glucagon (1–29) with a C-terminal extension Gly30,31Lys32-FITC (fluorescein isothiocyanate)], which has similar potency for cAMP as the native ligand [17], were obtained from Wuxi Apptec.

### 2.3 Cholesterol-modulating treatments

Cells were treated with cholesterol-saturated methyl-β-cyclodextrin (Sigma–Aldrich; referred to as “cholesterol” hereafter when in the context of cellular treatments; 50 μg/ml unless otherwise stated), or cholesterol-depleted methyl-β-cyclodextrin (Sigma–Aldrich; MβCD; 3 mM unless otherwise stated), in Hank's buffered saline solution (HBSS) for 30 min. Plated cells were treated with 10 μM simvastatin (Sigma–Aldrich) and/or 50 μM mevalonate (Sigma–Aldrich) for 16 h in serum-free medium. All treatments were washed off prior to experiments. Cell toxicity following treatments was measured using MTT Cell Viability Assay (Thermo Fisher), as per manufacturer's instructions.

### 2.4 cAMP accumulation

Huh7-GCGR cells plated in 96-well plates, or primary mouse or human hepatocytes in suspension, were stimulated with agonist in serum-free medium for 10 min at 37 °C. Cells were lysed, and cAMP was assayed by immunoassay (Cisbio HTRF cAMP Dynamic 2). For further details see Supplementary Methods.

### 2.5 cAMP detection by live cell imaging

Huh7-GCGR cells were transduced with cADDis biosensors [19] (Montana Molecular) in a BacMam vector; for further details see Supplementary Methods.

### 2.6 PKA activation assay

PKA activation was assessed in Huh7-GCGR cells by FRET imaging using AKAR4-NES [20] (gift from Jin Zhang, Addgene plasmid # 64,727); for further details see Supplementary Methods.

### 2.7 Mini-G NanoBRET assay

Recruitment of Mini-G_s_ and mini-G_i_ was assessed in Huh7-GCGR cells transfected with Mini-G constructs tagged with nanoluciferase [21] (a gift from Prof Nevin Lambert, Medical College of Georgia) and the membrane marker KRAS-venus; for further details see Supplementary Methods.

### 2.8 Molecular dynamics simulations, analysis and computational binding saturation curves

Structures of the GCGR in inactive and active conformations were derived from the Protein Data Bank (PDB ID: inactive 5XEZ, active 6LMK) [22,23]. Please see Supplementary Methods.

### 2.9 Hepatocyte glucose output assay

Primary mouse hepatocytes were serum-starved overnight with the addition of simvastatin 10 μM or vehicle (DMSO). After washing, phenol-red free DMEM with 1 mM sodium pyruvate (Gibco) and 20 mM sodium lactate (Sigma) was added. Baseline samples were taken before addition of glucagon 100 nM or vehicle, and following incubation at 37 °C for 24 h. Glucose was assayed using a glucose oxidase assay (Randox), normalised to protein content of the well (BCA assay, Thermo Fisher), and expressed as fold change of glucose production in the absence of glucagon.

### 2.10 Animals and dietary manipulation

Experiments were performed in accordance with the UK Animals (Scientific Procedures) Act 1986 and approved by the Animal Welfare and Ethical Review Board at Imperial College London. C57BL/6J male mice (Charles River) were group-housed in a pathogen-free facility at controlled temperature (22 °C) with a 12-hour light dark cycle. Access to food was *ad libitum*, except prior to fasting studies, and mice always had free access to water. Mice were fed standard chow (SDS Rm3) during acclimatisation. Specialist chows were based on a standard Clinton/Cybulsky rodent diet (10% kcal from fat and 70% kcal from carbohydrate) and were identical in constitution and calorie content, except for 0% (Control) or 0.5% added cholesterol (Chol), or 0.5% cholesterol and 120 mg/kg simvastatin (Chol/simva) (Research Diets). Mice were fed specialist diets for 7–9 days before glucagon challenge test and 12 days before the cull, via decapitation following a 5-hour fast. The liver was harvested, snap frozen in liquid nitrogen and stored at −80 °C.

### 2.11 Glucagon challenge test

Glucagon challenge tests were performed in the light phase, in mice fasted for 5 h [24,25]. Tail vein blood glucose was measured using a glucometer (Nexus, GlucoRx) before and at intervals following intraperitoneal injection with 2 g/kg sodium pyruvate (Sigma) as a gluconeogenic substrate ± 10 nmol/kg glucagon. Each mouse underwent the test with and without glucagon, in a random order, with an intervening washout period of 3 days.

### 2.12 Lipid extraction

Lipids were extracted from liver tissue by homogenization in ethanol (0.03x w/v) [26], and from cells by agitation in butanol, before evaporation and resuspension in methanol.

### 2.13 Biochemical assays

Liver triglyceride was measured using a GPO-PAP Triglyceride assay (Randox) and cholesterol using Amplex Red Cholesterol Assay Kit (Thermo Fisher). Serum glucagon and alanine were measured using commercial kits available from Cisbio and Sigma–Aldrich respectively.

### 2.14 Statistical analyses

All statistical analysis of experimental data was performed using Prism 9.2.0 for MacOS (Graphpad Software, San Diego, California USA). Concentration-response curves were generated by 3-parameter logistic fitting or using a “bell-shaped” fit. For cAMP, E_max_ and log_10_-transformed EC_50_ values were derived for each repeat and then compared using t-tests or one-way ANOVA, with matched analyses performed where permitted by experimental design, and multiple comparison tests as indicated in the figure legends. For experiments using pertussis toxin, the Gα_i_ component of the bell-shaped response was calculated by subtracting response in the presence of pertussis toxin (i.e. the Gα_s_-specific response) from total response. For cellular treatments, as a combined measure of agonism, E_max_/EC_50_ was calculated and normalised to vehicle control; log_10_-transformed values were then used for simple linear regression analysis, with calculation of goodness of fit. Glucagon-alanine index was calculated by multiplying fasting levels of glucagon (pmol/l) with alanine (mmol/l) [27]. p < 0.05 was considered statistically significant.

## 3 Results

### 3.1 Acute manipulation of cellular cholesterol content modulates GCGR signalling

**Figure 1:**
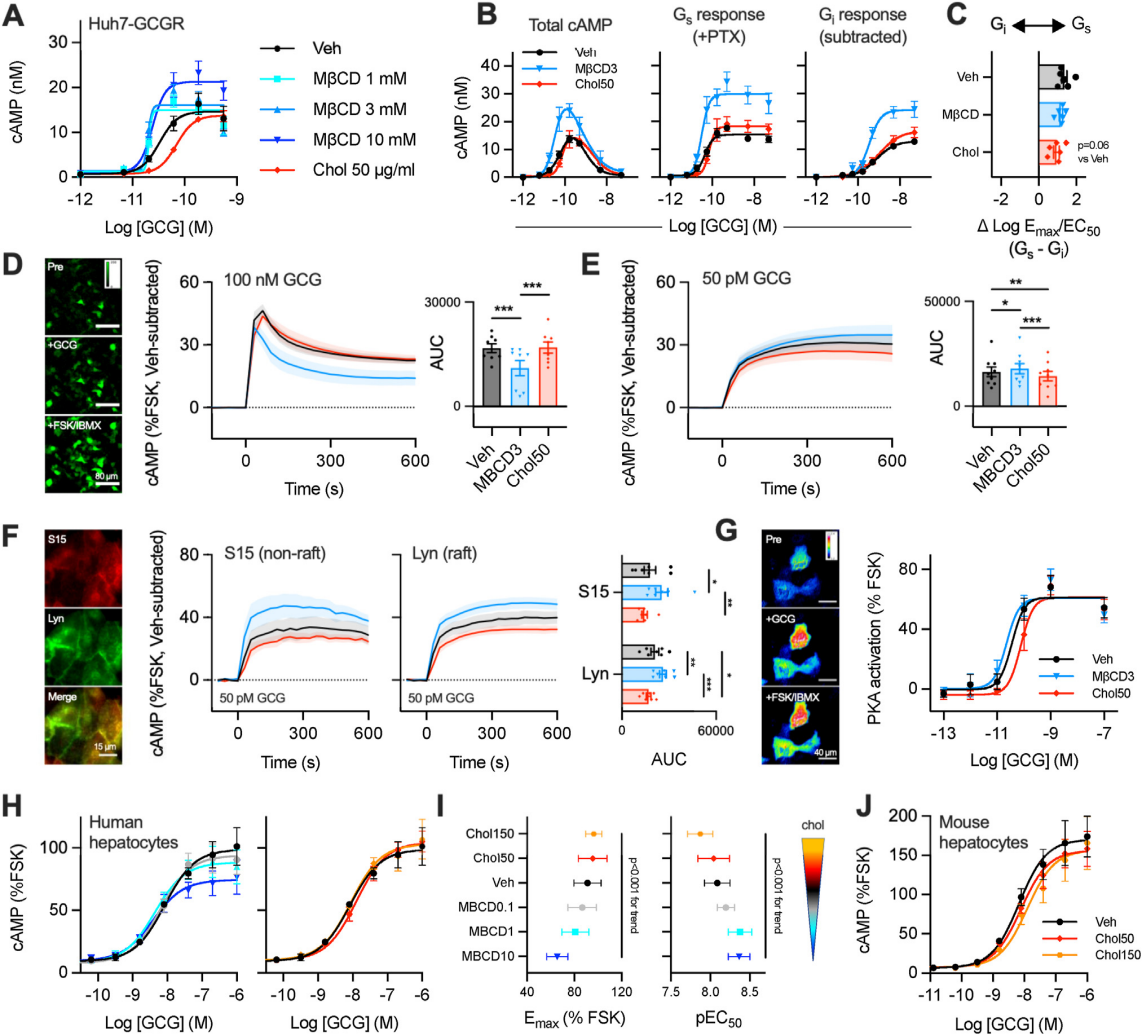
**Acute changes in cellular cholesterol levels influence glucagon-stimulated cAMP production in a concentration-dependent manner** (**A**) cAMP concentration-response curves in Huh7-GCGR cells pre-treated with cholesterol-deplete MβCD at indicated concentrations or cholesterol-saturated MβCD (Chol), then stimulated with glucagon (GCG), n = 4. (**B**) cAMP responses over a wider glucagon concentration range with or without pertussis toxin pre-treatment (PTX; 10 ng/ml) to block Gα_i_-mediated cAMP inhibition and reveal the Gα_s_- and Gα_i_-specific responses, n = 6. The effect of pre-treatment with MβCD or cholesterol is shown. (**C**) Balance between Gα_s_ and Gα_i_-mediated cAMP effects from (**B**); all inter-group statistical comparisons non-significant by one-way repeated measures ANOVA. (**D**) cAMP dynamics measured in Huh7-GCGR cells transduced with cADDis sensor (pictured; scale bar = 80 μm) pre-treated with MβCD or cholesterol and stimulated with 100 nM glucagon. Representative images from cells at baseline, 5 min after glucagon stimulation, and 5 min after addition of 100 μM IBMX and 10 μM forskolin (FSK). AUC comparison by one-way repeated measures ANOVA with Tukey's test, *n* = 8. (**E**) As for (D) but 50 pM glucagon, *n* = 9. (**F**) As for (D) but using membrane-targeted cADDis cAMP sensors as indicated, *n* = 7. (**G**) PKA activation in Huh7-GCGR cells expressing AKAR4-NES and pre-treated with MβCD or cholesterol. Representative FRET images of same cells at baseline, 5 min after glucagon stimulation, and 5 min after addition of 100 μM IBMX and 10 μM forskolin (FSK); scale bar 40 μm, *n* = 5. (**H**) cAMP accumulation in primary cadaveric human hepatocytes, 10 min stimulation with 100 μM IBMX, *n* = 4. The two panels are from the same experiments and separated for clarity. (**I**) Quantification of signalling potency and maximum responses from (H) in relationship to cholesterol modulating treatments, with one-way repeated measures ANOVA and linear test for trend. (**J**) cAMP accumulation in primary mouse hepatocytes, 10 min stimulation with 100 μM IBMX, *n* = 5. ∗p < 0.05, ∗∗p < 0.01, ∗∗∗p < 0.001. Data are shown as mean ± SEM, with individual experimental replicates where possible.

We first examined the impact of pharmacological modulation of cellular cholesterol levels on GCGR signalling in Huh7 hepatoma cells. GCGR was exogenously expressed due to undetectable endogenous levels [17]. At picomolar concentrations of glucagon, cAMP signalling was enhanced by pre-treatment with cholesterol-free methyl-β-cyclodextrin (MβCD), which rapidly extracts cholesterol from cellular membranes, whereas cholesterol loading with cholesterol-saturated MβCD had the opposite effect (Figure 1A; Supplementary Table 2). MβCD was not toxic to cells (Supplementary Figure 1A). GCGR shows a hormetic (bell-shaped) cAMP concentration-relationship due to superimposed effects of Gα_s_-mediated stimulation and Gα_i_-mediated inhibition of cAMP production [28], so we investigated the effects of modifying cellular cholesterol content on the Gα_s_ and Gα_i_ components of the overall cAMP response over a wide glucagon concentration range using the Gα_i_ inhibitor pertussis toxin [29] (Figure 1B). The Gα_i_ response required at least 10-fold more glucagon than the Gα_s_ response (Supplementary Table 1) but became dominant at higher concentrations. We found that both Gα_s_- and Gα_i_-mediated control of cAMP levels were increased by cholesterol depletion, and diminished by cholesterol loading, such that the overall balance between each component was not significantly altered (Figure 1C).

Given these opposing effects of Gα_s_ and Gα_i_ on GCGR signalling, we decided to investigate cAMP dynamics in Huh7-GCGR cells using a fluorescent biosensor, cADDis [19], which reports changes in intracellular cAMP in real time. At a supra-maximal glucagon concentration (100 nM; Figure 1D) a rapid peak in cAMP levels was followed by a steady reduction, whilst a much lower concentration (50 pM; Figure 1E) led to a sustained increase. Moreover, the effects of cholesterol manipulation on these responses were different at low and high glucagon concentrations, e.g. cholesterol depletion using MβCD increased the cAMP response at 50 pM but reduced it at 100 nM. This pattern is compatible with the net effect of MβCD reflecting the concentration-specific “dominant” Gα subtype response (i.e. Gα_s_ at lower concentrations, Gα_i_ at higher concentrations).

Membrane lipid composition is a key factor dictating how GPCRs and their intracellular effectors are concentrated into signalling nanodomains [16]. We therefore aimed to determine whether membrane cholesterol manipulation could alter the localisation of cAMP production in Huh7-GCGR cells using cADDis sensors targeted either to “raft” or “non-raft” membrane regions (Figure 1F). Whilst membrane cAMP production at 50 pM glucagon tended to be increased with MβCD and reduced with cholesterol loading, as was the case for total cellular cAMP, we did not find any difference between responses measured in each membrane sub-domain (Figure 1F). As a key mediator of GCGR-mediated liver effects downstream of cAMP [5], we also recorded cytosolic protein kinase A (PKA) activation using the FRET biosensor AKAR4 [30] (Figure 1G). The effects of cholesterol manipulation on PKA response potencies agreed with the pattern seen for cAMP (Supplementary Table 2).

GCGR ligand internalisation represents an additional downstream functional readout of GCGR activation. We examined this phenomenon in Huh7-GCGR cells following treatment with cholesterol-lowering or -enriching agents using fluorescein isothiocyanate-tagged glucagon (“FITC-GCG”), which closely mimics the pharmacology of native glucagon and has previously been used to study GCGR trafficking effects [17]. Interestingly, and in contrast to the effects on cAMP signalling, we observed an increase in FITC-GCG uptake after cholesterol loading, and a decrease after cholesterol depletion (Supplementary Figure 1B, 1C, Supplementary Table 1).

We next corroborated some of these findings using primary human hepatocytes from cadaveric donors (Figure 1H; Supplementary Table 2 for donor characteristics). Cholesterol depletion at increasing concentrations of MβCD progressively increased potency but reduced E_max_ for glucagon-induced cAMP, with cholesterol loading showing the opposite pattern (Figure 1I). cAMP signalling potency was also reduced by cholesterol loading in primary mouse hepatocytes (Figure 1J). Whilst the pronounced bell-shaped concentration–response relationship from Huh7-GCGR cells was not observed in either primary cell type, possibly as the phosphodiesterase inhibitor IBMX used in the primary hepatocyte assays promotes cAMP accumulation and would therefore reduce Gα_i_-dependent cAMP suppression, the pattern can again be explained by cholesterol levels influencing Gα_s_-dominant effects at low glucagon concentrations and Gα_i_ effects at higher glucagon concentrations. Although we did not measure it in our study, it is important to note that Gα_i_-dependent GCGR signalling effects extend beyond the suppression of cAMP production, for example leading to increases in c-Jun kinase (JNK) phosphorylation, a process which is implicated in GCGR-mediated hepatic glucose output [25].

### 3.2 Sustained cholesterol depletion using statins increases glucagon-induced hepatic glucose output

**Figure 2:**
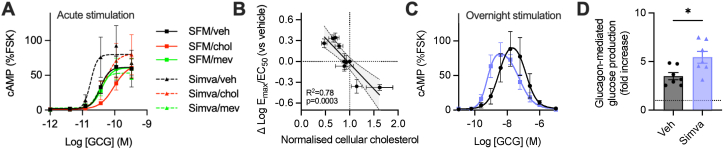
**Simvastatin treatment enhances glucagon-stimulated signalling and glucose output.** (**A**) The effect on glucagon-stimulated cAMP production (10 min) in Huh7-GCGR cells pre-treated with simvastatin (Simva) or serum-free medium (SFM) overnight, with concurrent or subsequent treatment with or without mevalonate (mev; 50 μM) or cholesterol-saturated MβCD (chol; 50 μg/ml). Results are normalized to forskolin (FSK; 10 μM), n = 5. (**B**) Association between a combined measure of cAMP efficacy and potency (log-transformed E_max_/EC_50_) and cellular cholesterol for each of the treatments shown in Figure 1A and Figure 2A, both normalized to vehicle control, with linear regression line ± 95% confidence intervals shown. (**C**) Steady state cAMP concentrations in Huh7-GCGR cells co-treated with glucagon and simvastatin/vehicle for 16 h, with normalisation to the acute 10 μM FSK response taken at the end of the incubation, *n* = 5. (**D**) Glucose production in primary mouse hepatocytes pre-treated with simvastatin or vehicle overnight, then stimulated with glucagon for 24 h, expressed as fold change over no-glucagon control stimulation, n = 7, with paired t-test. Data are shown as mean ± SEM, with individual experimental replicates in (D).

To investigate the potential for sustained reduction in cellular cholesterol to modulate GCGR signalling and establish a basis for how this might be relevant pharmacologically, we pre-treated Huh7-GCGR cells with the 3-hydroxy-3-methyl-glutaryl-coenzyme A reductase (HMGCR) inhibitor simvastatin to inhibit cholesterol synthesis. There was no cell toxicity with simvastatin treatment (Supplementary Figure 2A). Like MβCD-mediated acute cholesterol depletion, simvastatin also increased glucagon cAMP potency (Figure 2A, Supplementary Table 3); notably, this effect was reversed by supplementing cells with the HMGCR enzyme product mevalonate (which is otherwise depleted by statins), and also by acute restoration of membrane cholesterol levels using cholesterol-saturated MβCD (Figure 2A, Supplementary Table 3). There was a robust inverse correlation between relative cellular cholesterol content and cAMP responses from these assays and those presented in Figure 1A (see Figure 2B), whereas the opposite correlation was seen between cellular cholesterol and FITC-GCG uptake (Supplementary Figure 2B, 2C). In line with the effect of MβCD, both Gα_s_ and Gα_i_ cAMP responses were similarly augmented by simvastatin (Supplementary Figure 2D, 2E).

We also asked whether simvastatin could influence how hepatocytes respond to sustained periods of glucagon elevation, such as during fasting or with pharmacological GCGR agonism. After overnight stimulation with glucagon, inspection of steady state cAMP levels in Huh7-GCGR cells again suggested that both Gα_s_- and Gα_i_-dependent GCGR signalling are potentiated by simvastatin, as the entire bell-shaped concentration-response curve was shifted to the left (Figure 2C, Supplementary Table 3). Moreover, glucagon-induced glucose output from primary mouse hepatocytes was increased by simvastatin treatment (Figure 2D).

Overall, these data indicate that acute and sustained changes to cellular cholesterol levels *in vitro* can bidirectionally affect GCGR cAMP signalling, trafficking, and downstream effects.

### 3.3 Molecular dynamics simulations reveal potential GCGR-cholesterol binding sites

**Figure 3:**
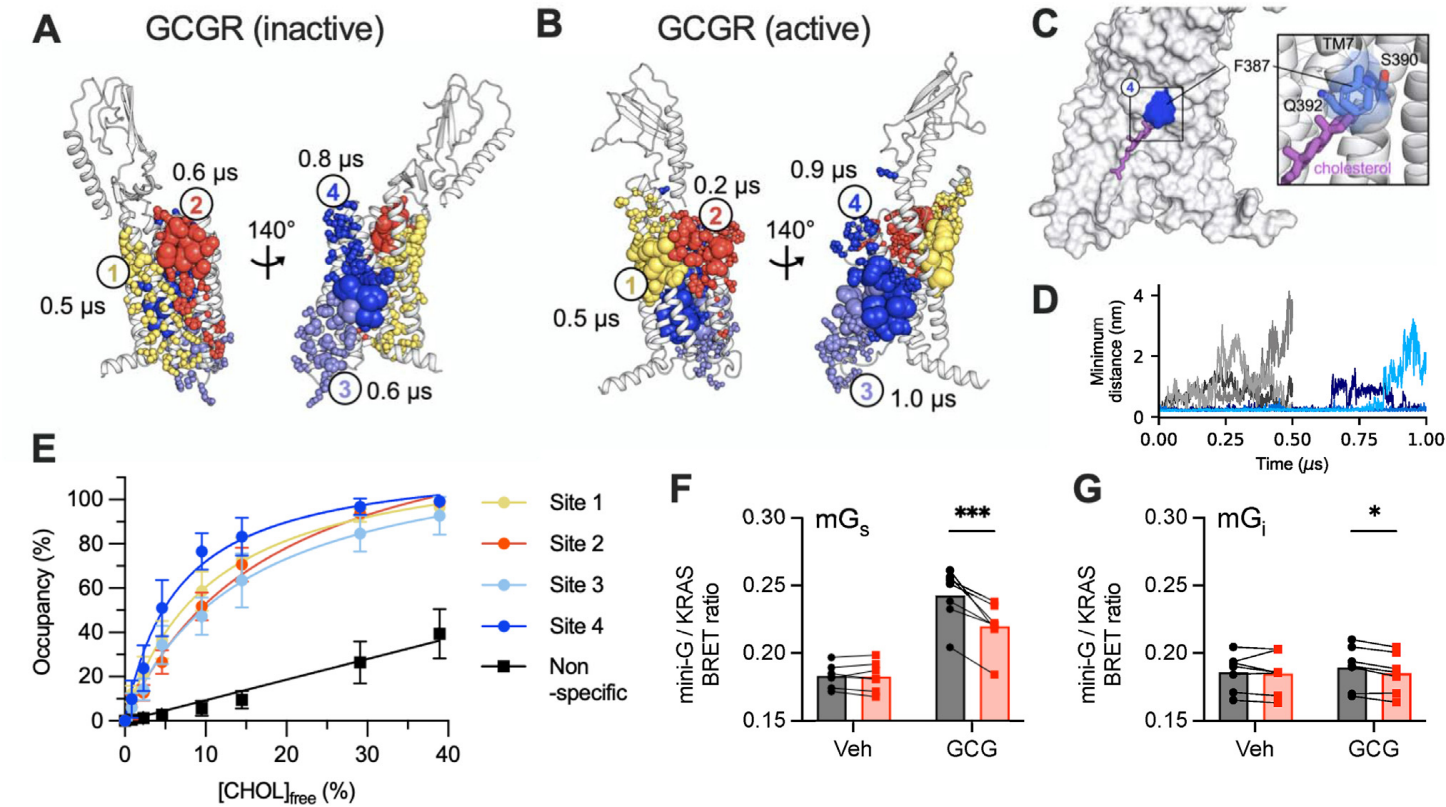
**Predicted GCGR-cholesterol interactions.** The top four ranked binding sites for cholesterol from coarse-grained MD simulations of the glucagon receptor (GCGR) in inactive (**A**) and active (**B**) conformations in plasma membrane-like bilayers containing 25% cholesterol. Each conformation was simulated for 10 × 10 μs. Distinct binding sites are coloured yellow (site-1), red (site-2), lilac (site-3) and blue (site-4). Residues comprising each site are shown as spheres scaled by per residue cholesterol residence times. The residence time for cholesterol binding to each site is indicated. Binding sites and associated residence times were calculated using PyLipID [43]. (**C**) Snapshot from atomistic simulations of the top ranked cholesterol binding pose at site-4, as identified by PyLipID from the coarse-grained simulations. GCGR is shown in surface representation and cholesterol is shown as sticks. F387 is coloured blue and encloses a pocket which shields the cholesterol hydroxyl group from the membrane (see inset for coordinating residues of TM7). (**D**) Minimum distance between the site-4 cholesterol and L395 (a key residue in site-4) across atomistic simulations. Simulations were initiated from the top ranked cholesterol pose whereby the hydroxy group was located towards the center of the bilayer (3 × 1 μs, blue) or with the cholesterol reversed by 180° such that the hydroxyl group was in proximity to the lipid phosphate groups (3 × 500 ns, grey). (**E**) Binding saturation curves for cholesterol binding to each site from equilibrium MD simulations (5 × 5 μs at each % free cholesterol). Site % occupancy was calculated using PyLipID and plotted against the free cholesterol % (see methods) in binary bilayers composed of POPC and cholesterol. (**F**) BRET signal (535/460) indicating interaction between nanoluciferase-tagged mini-G_s_ and GCGR in the plasma membrane in Huh7-GCGR cells expressing KRAS-venus, 30 min after stimulation with vehicle or 100 nM glucagon, *n* = 7, compared by two-way repeated measures ANOVA with Sidak's test. (**G**) As for (F) but using mini-G_i_. ∗p < 0.05, ∗∗∗p < 0.001. Data are shown as mean ± SEM, with individual experimental replicates in Figure 2F, G.

To explore the potential for direct interactions between the GCGR and cholesterol in the plasma membrane as an underlying mechanism for our pharmacological observations, we performed coarse-grained MD simulations of full-length GCGR within bilayers in active and inactive states. A single receptor molecule was simulated whilst embedded in plasma membrane mimetic bilayers containing 25% cholesterol. The locations of predicted cholesterol binding sites with the four highest cholesterol residence times were the same for the two GCGR conformations (Figure 3A, B, Supplementary Video 1). These correspond to a binding site between helices TM1 and TM2 (site-1), the extracellular portion of TM3/TM4 (site-2), an intracellular site formed by TM5, ICL3 and TM6 (site-3), and a densely packed site at the centre of TM6/TM7 (site-4). We note that for site-1, in the inactive GCGR conformation interacting residues were diffuse (Figure 3A), whereas in the active conformation they were restricted to the extracellular region (Figure 3B). The residues involved in formation of other sites were in broad agreement.

The following is the supplementary data related to this article:

Video 1 4 Video 1

For the active GCGR conformation, cholesterol binding sites in proximity to TM5/TM6 (site-3 and site-4) were stabilised and site-2 interactions were destabilised when compared to the inactive conformation. These findings indicate that, whilst the location of key cholesterol binding sites is comparable between full-length GCGR conformations, receptor activation induces subtle changes in cholesterol kinetics that result in more prolonged residence times around TM5, ICL3, TM6 and TM7. Surprisingly, the top ranked cholesterol pose at site-4 from the course-grained simulations was oriented with the β_3_-hydroxyl group at the bilayer midplane (i.e. flipped by 180° compared to the anticipated cholesterol orientation based on that within the bilayer) (Figure 3C). To examine this further we performed microsecond atomistic simulations of the site-4 cholesterol which remained stably bound for the majority of the trajectories (Figure 3D, blue), in agreement with the predicted residence time from coarse-grained simulations (0.8 μs). In contrast, control simulations initiated with cholesterol bound in the reverse (conventional, i.e. β_3_-hydroxyl group towards the bilayer/water interface) orientation at site-4 led to rapid cholesterol dissociation (<0.2 μs) (Figure 3D, grey). We attribute the stability of this unusual cholesterol pose at site-4 to H-bonding interactions between the cholesterol hydroxyl group and S390/Q392 on TM7 and due to F387 which folds over the top of the cholesterol molecule, effectively shielding the β_3_-hydroxy group from the surrounding hydrophobic membrane environment (Figure 3C).

To further evaluate the relevance of identified sites we performed simulations using membrane bilayers with varying cholesterol content [31] which allowed us to estimate apparent dissociation constants for cholesterol at each site (Figure 3E): K_d_^app^ at site-1: 10.7 ± 0.3%; site-2: 18.3 ± 0.4%; site-3: 15.0 ± 0.8%; and site-4: 6.6 ± 0.1%. The site-4 K_d_^app^ was comparable to ‘strong’ cholesterol binding sites observed on various other membrane proteins e.g. on the TRP channel Polycystin-2 (11 ± 1%), on Patched1 (6.8 ± 0.3%) and on the GPCR 5-HT_1A_ (4–9%) where cholesterol densities have been observed in structures and cholesterol is implicated in biological function [31]. While interpreting the relative specificity of cholesterol sites from kinetics alone can be challenging, saturable binding curves were observed which were distinct from background non-specific interactions (Figure 3E, black line). Thus, predicted affinities for cholesterol interactions with GCGR were comparable to those for ‘strong’ cholesterol sites on other proteins, which in turn correlates with the results of our biochemical experiments.

Site-3 overlaps with the experimentally verified G protein binding site for GCGR and other class B GPCRs [23,32]; and site-4 corresponds to TM6, which tilts outwards during receptor activation to accommodate G protein binding. In view of the inverse correlation between membrane cholesterol content and cAMP signalling (Figure 2B), we hypothesised that cholesterol could act as a negative allosteric regulator at sites-3/4 [33] by reducing the capacity of the receptor to interact with Gα_s._ We investigated this possibility by measuring recruitment of mini-G_s_, a conformational biosensor for Gα_s_-favouring active GPCR conformations [34], and found that cholesterol loading reduced glucagon-stimulated mini-G_s_ recruitment to plasma membrane GCGR in Huh7-GCGR cells (Figure 3F). Whilst the ligand-induced mini-G_i_ recruitment response was much smaller than for mini-G_s_, as previously shown in HEK293 cells [35], this was also reduced by cholesterol loading (Figure 3G). These observations are in line with our cAMP data in Figure 1B indicating the PTX-sensitive and insensitive elements of the GCGR cAMP response are both suppressed by cholesterol enrichment.

### 3.4 Increasing hepatic cholesterol in mice decreases glucagon sensitivity

**Figure 4:**
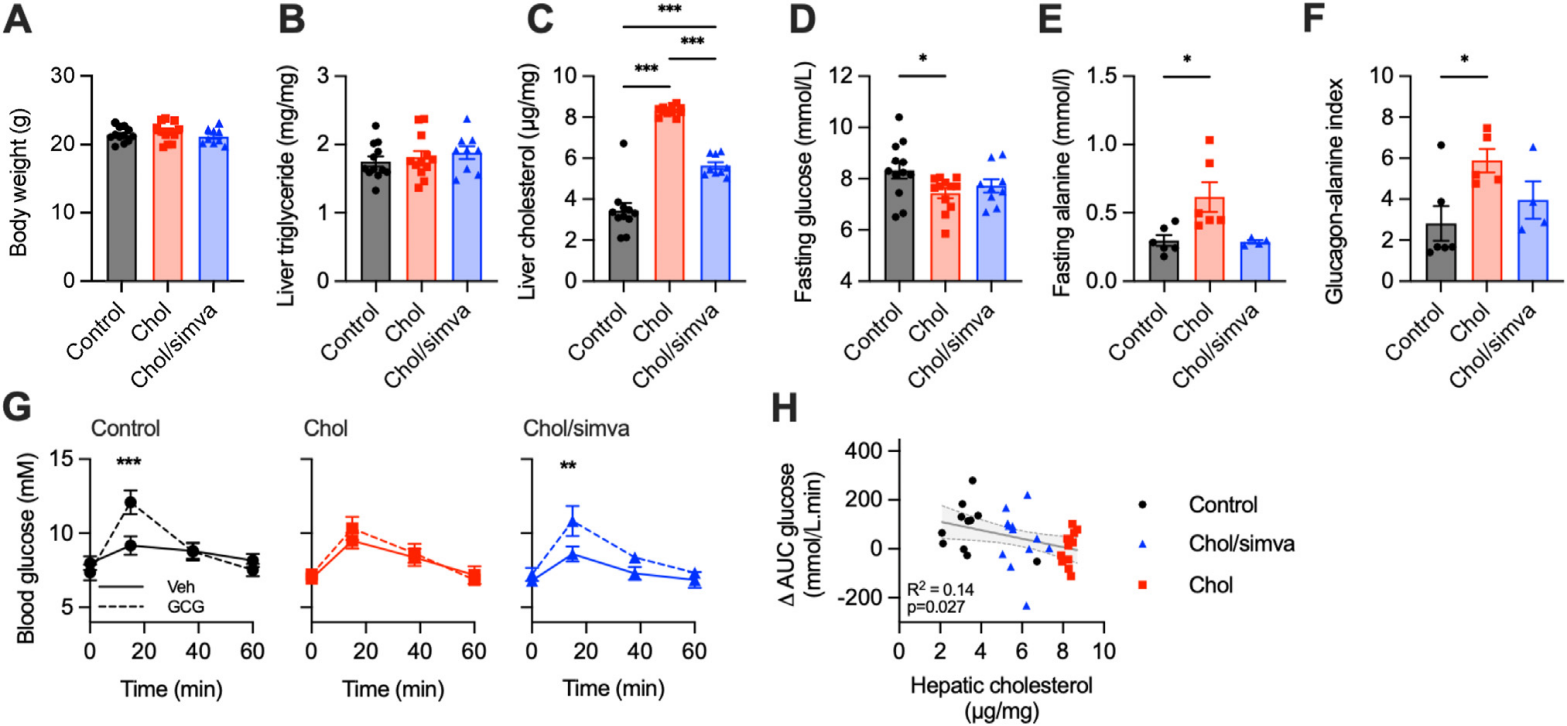
**Increase in hepatic cholesterol in mice decreases responsiveness to glucagon.** (**A**) body weight, *n* = 9–12, (**B**) hepatic triglyceride, *n* = 9–12, (**C**) hepatic cholesterol, *n* = 9–12, (**D**) fasting glucose, *n* = 9–12, (**E**) fasting alanine, *n* = 6, and (**F**) glucagon:alanine index, *n* = 6, in mice fed different diets for 7–12 days; statistical comparisons are by one-way ANOVA with Tukey's test. (**G**) glucagon/pyruvate challenge test, *n* = 12, with blood glucose compared using two-way repeated measures ANOVA with Sidak's test. (**H**) the association between change in AUC during glucagon challenge test and hepatic cholesterol in mice fed different diets as indicated by colour code; the linear regression line ± 95% confidence intervals is shown. ∗p < 0.05, ∗∗p < 0.01, ∗∗∗p < 0.001. Data are shown as mean ± SEM, with individual experimental replicates where possible.

To explore the potential relevance of these findings *in vivo*, we next manipulated hepatic cholesterol in adult mice using isocaloric chow supplemented with or without 0.5% cholesterol (chol) with or without simvastatin (chol/simva). Whilst these diets had no effect on body weight or hepatic triglyceride content (Figure 4A, B), the cholesterol-enriched diet caused a dramatic increase in hepatic cholesterol after 1 week, which was partially abrogated by simvastatin (Figure 4C). Although fasting glucagon was comparable between groups (control: 9.2 ± 1.9 pmol/L; chol 12.0 ± 1.9 pmol/L; chol/simva 16.1 ± 2.6 pmol/L, ns by one-way ANOVA with Tukey's test), mice fed a high cholesterol diet had a lower fasting plasma glucose, consistent with reduced glucagon signalling [36,37] (Figure 4D). We also measured fasting alanine in a subset of mice, which is expected to be high when glucagon receptor signalling is impaired due to the role of glucagon in amino acid catabolism [27,38], and found that mice fed high cholesterol diet had higher fasting alanine and glucagon-alanine index than control mice (Figure 4E, F). Notably, these changes were abrogated in mice also fed simvastatin.

We performed an intra-peritoneal glucagon/pyruvate challenge test after 5 h of fasting, at which point both glycogenolysis and gluconeogenesis (with pyruvate providing the substrate for the latter) can be stimulated by glucagon. Here, the expected glucagon-induced peak in glycaemia was absent in mice that had been fed the high cholesterol diet, but partly restored in mice fed the cholesterol with simvastatin diet (Figure 4G). We noted an inverse association between hepatic cholesterol and the glucose excursion induced by glucagon (Figure 4H).

Therefore, in keeping with our *in vitro* data, increased hepatic cholesterol results in diminished GCGR signalling in response to both endogenous and exogenous glucagon.

## 4 Discussion

In this study we demonstrate an inverse relationship between hepatocyte cholesterol and glucagon responsiveness, as measured *in vitro* by glucagon-stimulated cAMP and glucose production, and *in vivo* from fasting metabolic parameters and the hyperglycaemic response to exogenous glucagon. Further, we have identified probable cholesterol binding sites on GCGR that could mediate these effects.

To our knowledge this is the first time the effect of cholesterol on GCGR function has been reported. Increasing membrane cholesterol enhances the function of some GPCRs, e.g. the α_1A_-adrenergic receptor, and diminishes that of others, e.g. the cannabinoid receptor 1 and β_1_-adrenergic receptor [33,39]. We previously showed that cholesterol depletion in pancreatic beta cells led to reductions in both cAMP signalling and ligand-induced endocytosis of the closely related GLP-1R [16]. This partly contrasts with our current GCGR results, in which cholesterol depletion reduced uptake of fluoresecent glucagon but enhanced potency for Gα_s_-dependent cAMP production. The discrepancy may reflect inherent differences in the effect of cholesterol on the two receptors, though it is worth noting that GPCR function is also modulated by concomitantly-interacting membrane proteins, e.g. Receptor Activity Modifying Protein 2 (RAMP2) [17], and other membrane constituents that differ depending on cell type, which could in turn impact the role played by cholesterol. Other class B GPCRs for which the impact of cholesterol manipulation have been studied are summarised in Supplementary Table 4.

Somewhat peculiar to GCGR signalling are the superimposed and opposing effects of Gα_s_ and Gα_i_ on cAMP production, which led to a pronounced bell-shaped concentration–response relationship for cAMP. As the Gα subtype “preference” varied across the glucagon concentration range, and both components were increased when cholesterol was depleted or decreased on cholesterol loading, the net effect of cholesterol manipulation on cAMP levels was concentration-specific. It is important to note that, even though cAMP is a useful indicator of how Gα_s_ and Gα_i_ effects are shaped by changes to cellular cholesterol, the impact on downstream readouts may not show the same hormetic relationship. For example, 100 nM glucagon caused marked suppression of cAMP production relative to lower concentrations, but did not suppress PKA activation to the same extent, likely reflecting the redundancy inherent to many intracellular signalling systems. Moreover, both Gα_s_ and Gα_i_ signalling are known to actively contribute to GCGR effects on hepatic glucose output despite their opposite effects on cAMP, with JNK implicated as a key intermediate in the transduction of Gα_i_ activation in this context [25]. We also observed cholesterol-mediated increases in GCGR internalisation, which is likely to have complex roles in signalling through regulating the availability of surface receptors e.g. via promoting lysosomal degradation, but also in providing a platform for sustained endosomal cAMP generation and engagement with spatially constrained signalling networks not accessible to membrane-resident receptors [40].

There are various mechanisms by which cholesterol and other lipids may alter the stability, ligand binding properties, and thus function of GPCRs [10]: direct competition with agonist binding at the orthosteric site; directly binding at an allosteric site to modulate receptor conformation and dynamics [33]; indirectly via a change in local membrane composition and properties; or a combination of the above e.g. due to interplay between orthosteric and allosteric sites [39]. Recent work has demonstrated that GCGR function can be affected by endogenous allosteric modulators [17,41]. GCGR has computationally predicted potential allosteric cholesterol binding sites, but the validity of these so-called “CRAC” and “CARC” consensus motifs in general has been questioned [42]. Our recently developed MD simulation method to evaluate GPCR-lipid interactions [43] has allowed us to identify probable binding sites for cholesterol on the GCGR, with residence times which differ slightly depending on the receptor state. Site-4, which has the longest cholesterol residence time and lowest K_d_^app^, overlays with the observed binding of a negative allosteric regulator [22] and antagonist [44] in inactive GCGR structures. Cholesterol has been observed to bind to sites-1/2/3 in the structures of other G protein bound class B1 GPCRs, however site-4 is not observed in active-like conformations (Supplementary Table 5). Thus, we propose cholesterol binding to site-4 may be responsible for our observed decrease in glucagon responsiveness and mini-G_s_ binding by shifting the population ensemble toward the inactive conformation [14]. Additionally it is possible that lipid synergy or membrane biophysical properties may further regulate receptor signalling. For example sites-3/4 are in proximity to previously observed PIP_2_ binding sites on GCGR [45]. Lipid interplay has been suggested to occur for anionic lipid binding to the ion channel Kir2.2 [46] and warrants further investigation in GPCRs.

An alternative mechanism for GCGR regulation may be receptor redistribution, for example retention intracellularly [47], or into distinct lipid nanodomains. Changes to the pool of accessible cholesterol within sphingomyelin enriched regions [48], for example as a result of diet [49,50], may alter the combination of occupied cholesterol sites. Our observed K_d_^app^s are within the (patho)-physiological range of membrane cholesterol, rendering differential GCGR partitioning between lipid pools due to changes in cholesterol binding/unbinding physiologically feasible. Using targeted biosensors to measure cAMP production in lipid raft and non-raft membrane region we did not find any evidence that the localisation of cAMP production was preferentially modulated by cholesterol modulating treatments; however, direct monitoring of receptor/effector redistribution would be required to fully investigate this possibility.

GCGR activation causes increased hepatic glucose production and, corresponding with our *in vitro* study results, we observed that a high cholesterol diet reduced the hyperglycaemic response to a glucagon stimulus in mice, and that this effect was partially reduced by simvastatin treatment. The effect observed was small, possibly due to compensatory mechanisms that make the investigation of glucagon sensitivity challenging *in vivo* [5]. Supporting this observation however, we observed an increase in surrogate markers of glucagon resistance (fasting serum alanine and glucagon-alanine index) in mice with high hepatic cholesterol, along with relative fasting hypoglycaemia. Glucagon resistance has previously been reported in patients with obesity and NAFLD [3,6] but here we demonstrate glucagon resistance in mice fed a cholesterol-rich diet for just seven days, a period sufficiently short to result in no alteration in body weight nor hepatic fat content. There are intriguing implications of these physiological findings for human health and disease. Statin treatment is associated with increased incidence and worsening of established T2D [[51], [52], [53]], and the degree of low-density lipoprotein-cholesterol (LDL-C) reduction correlates with the likelihood of developing T2D [52]. Genetic polymorphisms of *HMGCR* and related genes that reduce LDL-C also increase the probability of developing T2D [54,55], whereas patients with monogenic familial hypercholesterolaemia have high levels of hepatic cholesterol and a reduced risk of incident T2D [56,57]. Our data suggest that a reduction in hepatocyte membrane cholesterol may contribute to these effects by increasing sensitivity to the hyperglycaemic effects of physiological glucagon. Our data also potentially reconcile the observations that NAFLD is associated with both an increase in hepatic cholesterol [7,8] and glucagon resistance [6,58]. In NAFLD, glucagon resistance has been proposed to drive impaired glucose tolerance and T2D in a subset of patients. This may be via perturbation of the alpha cell–hepatocyte axis, whereby hepatocytes resistant to glucagon are less able to catabolise circulating amino acids, which causes hypersecretion of glucagon by alpha cells [59]. In this context, hyperglucagonaemia is still capable of increasing glycaemia as glucagon resistance is incomplete. In view of these ostensibly opposite effects on glycaemia that could result from cholesterol-mediated glucagon resistance, further studies are needed to carefully examine which process dominates in different pathological states. Nevertheless, greater understanding of this relationship is likely to underpin new therapies for NAFLD [60].

Antagonising glucagon signalling has long been proposed as a therapeutic strategy for T2D [61]. Our data suggest that cholesterol binding site-4 could be targeted by small molecule allosteric modulators of GCGR activity, as for other GPCRs [62]. Conversely, GCGR agonism is increasingly seen as a viable component of multi-incretin treatment for obesity and diabetes as, when combined with GLP-1R agonism, beneficial effects of glucagon (e.g. amelioration of hepatic steatosis and increased energy expenditure) may be realised without unwanted hyperglycaemia [63]. Our work suggests that it is worth evaluating whether lipid-modifying treatments can increase effectiveness of therapeutic GCGR agonism in metabolic disease.

Our study has limitations. The experimental approaches we used to manipulate cholesterol each have caveats. MβCD is likely to sequester additional lipids from the plasma membrane [64], which could themselves influence receptor function [45], although our study benefits from using lower MβCD concentrations than many others (see Supplementary Table 4). Inhibition of HMGCR with statins reduces synthesis of not only cholesterol but also of intermediaries required for post-translational protein modifications including farnesylation and geranylgeranylation [65]. Model lipid membrane experiments confirm that simvastatin decreases the cholesterol content of the membrane [66] but cannot exclude the possibility that they also modify other membrane constituents. Hepatoma cells are convenient for testing proximal GCGR signalling events such as cAMP and PKA activation, but are not suitable for modelling downstream metabolic responses such as glucose output or β-oxidation. Beyond confirming the effect of simvastatin on glucagon-induced hepatic glucose output from mouse hepatocytes, our manuscript lacks an in-depth evaluation of these phenomena in primary cell models, which is essential to fully understand the implications of the upstream signalling responses observed and should be considered for future work. The dietary changes we implemented in mice altered hepatic cholesterol content, but this is unlikely to have been plasma membrane specific. We also did not check for changes in surface GCGR expression in our *in vivo* model, and note that this can be reduced by high fat feeding [47]. Despite these caveats, we observed congruent results across different systems that support a role for cholesterol in the regulation of GCGR sensitivity, although it remains possible that different mechanisms underpin our results observed *in vitro* and *in vivo* despite them being directionally consistent. Furthermore, it remains unclear whether manipulating the lipid environment of the GCGR influences its signalling properties primarily via a direct effect on receptor function, or by altering the spatial dynamics of GCGR relative to its intracellular effectors: this could be investigated using membrane fractionation techniques, *in vitro* reconstituted systems, imaging approaches to co-visualise the receptor and its potential interactors, and solid-state NMR experiments at variable cholesterol concentrations.

To conclude, our data suggest that increased hepatocyte membrane cholesterol directly contributes to glucagon resistance and provide a potential molecular basis for this phenomenon.
