# Supplementary material for: Hepatocyte cholesterol content modulates glucagon receptor signalling
Source: Mol Metab. 2022 Jun 16;63:101530. doi: 10.1016/j.molmet.2022.101530 (PMC9254120; doi:10.1016/j.molmet.2022.101530)
Supplement: Multimedia component 1 [file mmc1.docx]

**Supplementary Figures**

**Hepatocyte cholesterol content modulates glucagon receptor signalling**

Emma Rose McGlone^1,2^, T. Bertie Ansell^3^, Cecilia Dunsterville^1^, Wanling Song^3,5^, David Carling^4^, Alejandra Tomas^1^, Stephen R Bloom^1^, Mark S. P. Sansom^3,*^, Tricia Tan^1^, Ben Jones^1,*^.

^1^ Department of Metabolism, Digestion and Reproduction, Imperial College London, London W12 0NN, United Kingdom.

^2^ Department of Surgery and Cancer, Imperial College London, London W12 0NN, United Kingdom.

^3^ Department of Biochemistry, University of Oxford, Oxford OX1 3QU, United Kingdom.

^4^ Cellular Stress Research Group, MRC London Institute of Medical Sciences, Imperial College London, London W12 0NN, United Kingdom.

^5^ Current address: Rahko, Clifton House, 46 Clifton Terrace, Finsbury Park, London N4 3JP, United Kingdom.

* Corresponding authors

**Contents:**

- Supplementary Figure 1
- Supplementary Figure 2


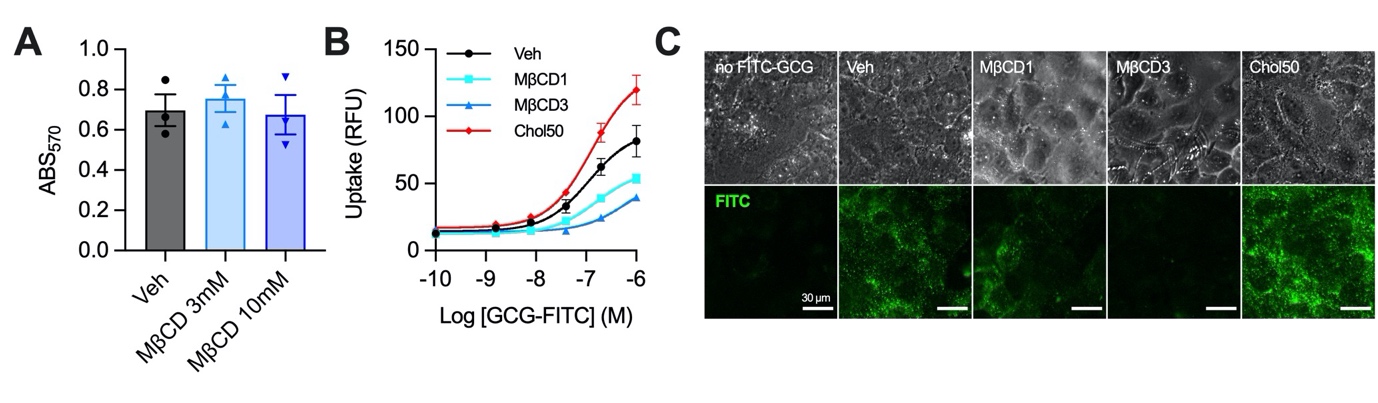


**Supplementary Figure 1:** (**A**) Cell viability as measured by absorbance following MMT assay in Huh7-GCGR cells pre-treated with cholesterol-deplete MβCD or vehicle, n=3. **(B)** The effect of pre-treatment with MβCD or cholesterol on FITC-GCG uptake in Huh7-GCGR cells, n=5. (**C**) Representative phase contrast (Ph) and FITC-epifluorescence images shown of experiments from (B), scale bar = 30 µm.

**Supplementary Figure 2:** (**A**) Cell viability as measured by absorbance following MMT assay in Huh7-GCGR cells pre-treated with 10 µM simvastatin for 16 hours, n=3. (**B**) The effect of pre-treatment with simvastatin ± mevalonate ± cholesterol, n=4, on FITC-GCG uptake in Huh7-GCGR cells, (**C**) Association between FITC-GCG uptake at 200 nM and cellular cholesterol content for the treatments shown in Supplementary Figures 1B and 2B, with linear regression line ± 95% confidence intervals shown. (**C**) cAMP responses with or without 10 ng/ml PTX pre-treatment to reveal the Gα_s_- and Gα_i_-specific responses, n=6. (**D**) Balance between Gα_s_ and Gα_i_-mediated cAMP effects from (C), non-significant by paired t-test.
