# Supplementary material for: Hepatocyte cholesterol content modulates glucagon receptor signalling
Source: Mol Metab. 2022 Jun 16;63:101530. doi: 10.1016/j.molmet.2022.101530 (PMC9254120; doi:10.1016/j.molmet.2022.101530)
Supplement: Multimedia component 2 [file mmc2.docx]

**Supplementary Tables**

**Hepatocyte cholesterol content modulates glucagon receptor signalling**

Emma Rose McGlone^1,2^, T. Bertie Ansell^3^, Cecilia Dunsterville^1^, Wanling Song^3,5^, David Carling^4^, Alejandra Tomas^1^, Stephen R Bloom^1^, Mark S. P. Sansom^3,*^, Tricia Tan^1^, Ben Jones^1,*^.

^1^ Department of Metabolism, Digestion and Reproduction, Imperial College London, London W12 0NN, United Kingdom.

^2^ Department of Surgery and Cancer, Imperial College London, London W12 0NN, United Kingdom.

^3^ Department of Biochemistry, University of Oxford, Oxford OX1 3QU, United Kingdom.

^4^ Cellular Stress Research Group, MRC London Institute of Medical Sciences, Imperial College London, London W12 0NN, United Kingdom.

^5^ Current address: Rahko, Clifton House, 46 Clifton Terrace, Finsbury Park, London N4 3JP, United Kingdom.

* Corresponding authors

**Contents:**

- Supplementary Table 1: Donor characteristics for human hepatocytes.
- Supplementary Table 2: Glucagon responses following acute cellular cholesterol manipulation.
- Supplementary Table 3: Glucagon responses in Huh7-GCGR cells following overnight treatments to manipulate cellular cholesterol.
- Supplementary Table 4: Class B GPCRs for which the impact of membrane cholesterol modulation has been reported.
- Supplementary Table 5: Class B GPCRs with deposited cholesterol binding sites on RCSB Protein Data Bank.
- Supplementary table references

**Supplementary Table 1: Donor characteristics for human hepatocytes.** Serology was performed for Hepatitis B, Hepatitis C and HIV.

| **Batch number** | **Age (years)** | **Gender** | **Race** | **Diabetes** | **Serology** |
| --- | --- | --- | --- | --- | --- |
| 1 | 64 | Male | Caucasian | No | Negative |
| 2 | 50 | Male | Unknown | No | Negative |
| 3 | 66 | Male | Caucasian | No | Negative |
| 4 | 66 | Male | Caucasian | No | Negative |

**Supplementary Table 2: Glucagon responses following acute cellular cholesterol manipulation.** All Huh-7-GCGR cells except for final row (mouse hepatocytes). Parameter estimates ± SEM from responses depicted in Figure 1, Supplementary Figure 1 and Supplementary Figure 2 as indicated. For uptake assays, E_max_ and EC_50_ are derived from pooled data before statistical analysis. Mean ± SEM; *n*=4 or 5. Uptake at a single concentration of FITC-GCG (200 nM) is presented because E_max_ and EC_50_ could not be derived from the curves for overnight treatments. Treatment effects were analysed using matched 1-way ANOVA and compared to vehicle with Dunnett’s multiple comparison test, or by paired t-test where there are only 2 groups. “n.d.” indicates not done. *p<0.05; **p<0.01; ***p<0.001.

|  | | Vehicle | MβCD (1 mM) | MβCD (3 mM) | MβCD (10 mM) | Cholesterol (50 µg/ml) | Cholesterol (150 µg/ml) |
| --- | --- | --- | --- | --- | --- | --- | --- |
| **cAMP**  **(Fig 1A)** | Log EC_50_ (M) | -10.5 ± 0.0 | -10.7 ± 0.0 * | -10.8 ± 0.1 * | -10.6 ± 0.1 | -10.2 ± 0.0 * |  |
|  | E_max_ (nM cAMP) | 14.9 ± 2.7 | 15.0 ± 1.2 | 16.1 ± 0.5 | 21.9 ± 2.4 | 14.8 ± 1.6 |  |
| **Gα_s_ (Fig 1B)** | Log EC_50_ (M) | -10.4 ± 0.1 | n.d. | -10.5 ± 0.1 | n.d. | -10.0 ± 0.1 ** |  |
|  | E_max_ (nM cAMP) | 15.4 ± 1.0 | n.d. | 31.3 ± 3.0 *** | n.d. | 19.6 ± 2.0 |  |
| **Gα_i_ (Fig 1B)** | Log EC_50_ (M) | -9.1 ± 0.1 | n.d. | -9.4 ± 0.2 | n.d. | -9.2 ± 0.3 |  |
|  | E_max_ (nM cAMP) | 13.4 ± 1.0 | n.d. | 26.1 ± 3.0 *** | n.d. | 16.6 ± 2.0 |  |
| **PKA (Fig 1G)** | Log EC_50_ (M) | -10.5 ± 0.1 | n.d. | -10.6 ± 0.1 | n.d. | -10.1 ± 0.1 * |  |
|  | E_max_ (% FSK) | 63.6 ± 7.4 | n.d. | 61.7 ± 4.7 | n.d. | 58.5 ± 5.2 |  |
| **GCG-FITC uptake (Fig S1B)** | Log EC_50_ (M) | -6.9 ± 0.2 | -6.8 ± 0.1 | -6.2 ± 0.2 ** | n.d. | -6.9 ± 0.1 |  |
|  | E_max_ (RFU) | 89.4 ± 8.0 | 60.1 ± 3.0 * | 56.5 ± 8.4 * | n.d. | 133.0 ± 8.0 ** |  |
|  | 200 nM (RFU) | 62.5 ± 6.3 | 38.8 ± 2.0 * | 24.6 ± 1.8 *** | n.d. | 87.8 ± 7.0 ** |  |
| **cAMP (mouse hepatocytes – Fig 1J)** | Log EC_50_ (M) | -8.2 ± 0.1 | n.d | n.d | n.d | -8.1 ± 0.1 | -7.9 ± 0.1 ** |
|  | E_max_ (% FSK) | 170.5 ± 28.0 | n.d | n.d | n.d | 156.9 ± 22.0 | 157.8 ± 37.3 |

**Supplementary Table 3: Glucagon responses in Huh7-GCGR cells following overnight treatments to manipulate cellular cholesterol.** Parameter estimates ± SEM from responses depicted in Figure 2 and Supplementary Figure 2. For uptake assays, E_max_ and EC_50_ are derived from pooled data before statistical analysis. Mean ± SEM; *n*=4 or 5. Uptake at a single concentration of FITC-GCG (200 nM) is presented because E_max_ and EC_50_ could not be derived from the curves for overnight treatments. Treatment effects were analysed using matched 1-way ANOVA and compared to vehicle with Dunnett’s multiple comparison test, or by paired t-test where there are only 2 groups. “n.d.” indicates not done. *p<0.05; **p<0.01; ***p<0.001.

|  | | SFM | SFM + Chol | SFM + Mev | Simva | Simva + Chol | Simva + Mev |
| --- | --- | --- | --- | --- | --- | --- | --- |
| **Acute stimulation cAMP (Fig 2A)** | Log EC_50_ (M) | -10.5 ± 0.1 | -10.0 ± 0.1 * | -10.3 ± 0.0 | -10.7 ± 0.1 * | -10.3 ± 0.1 | -10.5 ± 0.0 |
|  | E_max_ (% FSK) | 61.4 ± 14.1 | 69.0 ± 18.6 | 62.8 ± 16.0 | 83.8 ± 24.3 | 82.5 ± 29.9 | 50.9 ± 14.9 |
| **Sustained stimulation cAMP (Fig 2C)** | Stimulatory Log EC_50_ (M) | -8.5 ± 0.5 | n.d. | n.d. | -9.2 ± 0.2 | n.d. | n.d. |
|  | Inhibitory Log EC_50_ (M) | -6.9 ± 0.5 | n.d. | n.d. | -7.3 ± 0.3 | n.d. | n.d. |
| **Gα_s_ (Fig S2D)** | Log EC_50_ (M) | -10.2 ± 0.1 | n.d. | n.d. | -10.4 ± 0.1 *** | n.d. | n.d. |
|  | E_max_ (% FSK) | 84.0 ± 11.5 | n.d. | n.d. | 98.6 ± 12.4 | n.d. | n.d. |
| **Gα_i_ (Fig S2D)** | Log EC_50_ (M) | -9.5 ± 0.1 | n.d. | n.d. | -9.5 ± 0.3 | n.d. | n.d. |
|  | E_max_ (% FSK) | 73.7 ± 11.4 | n.d. | n.d. | 90.9 ± 14.0 | n.d. | n.d. |
| **GCG-FITC uptake (S2B)** | 200 nM (RFU) | 78.5 ± 13.4 | 98.6 ± 17.7 | 85.7 ± 10.5 | 89.2 ± 12.8 | 85.1 ± 19.2 | 79.8 ± 6.9 |

**Supplementary Table 4**: **Class B GPCRs for which the impact of membrane cholesterol modulation has been reported.**

| **Class B GPCR** | **Cell model** | **Manipulation and effect** |
| --- | --- | --- |
| Calcitonin gene-related peptide receptor (CGRPR) | Guinea pig gall bladder [1] smooth muscle | Cholesterol-saturated MβCD: ↓ CGRP-induced action potentials |
| Glucagon-like peptide receptor 1 (GLP-1R) | INS-1 cells [2] | 10 mM MβCD: ↓ exendin-4-induced cAMP efficacy and endocytosis |
|  | HEK293 cells [3] | 10 mM MβCD: ↓ exendin-4-C16-induced cAMP potency and endocytosis |
| Glucagon-like peptide 2 receptor (GLP-2R) | DLD-1 and BHK cells [4] | 10 mM MβCD: ↓ GLP-2-induced endocytosis, no effect on cAMP |
| Glucagon receptor (GCGR) | Huh7 cells and mouse hepatocytes (this work) | 1 – 10 mM MβCD, statin treatment: ↑ glucagon-induced cAMP, ↓ endocytosis; opposite effect from cholesterol loading |
| Pituitary adenylate cyclase–activating polypeptide type 1 receptor (PAC_1_R) | PC12 [5] | 10 mM MβCD: ↓ PACAP-38 cAMP efficacy |
| Parathyroid hormone type 1 receptor (PTHR1) | HEK293 cells [6] | 2% MβCD: no effect on PTH-induced augmentation of carbochol-induced Ca^2+^ transients |
| Vasoactive intestinal polypeptide receptor 2 (VPAC_2_R) | Mouse gastric smooth muscle cells [7] | 10 mM MβCD: ↓VIP-induced endocytosis |

**Supplementary Table 5**: **Class B GPCRs with deposited cholesterol binding sites on RCSB Protein Data Bank (**[**https://www.rcsb.org**](https://www.rcsb.org)**; accessed 13^th^ October 2021).**

| **Class B GPCR** | **Structures with modelled cholesterol** | **Structures with cholesterol bound at site-1, -2 or -3** |
| --- | --- | --- |
| Parathyroid hormone type 1 receptor (PTHR1) | 6NBF, 6NBI, 6NBH [8] | 6NBF (site-2), 6NBH (site-2, site-3) |
| Glucagon-like peptide receptor 1 (GLP-1R) | 7DUQ, 7E14 [9] | 7DUQ (site-1, site-3), 7E14 (site-3) |
| Corticotrophin-releasing factor 1 and 1 receptors (CRF1R and CRF2R) | 6PB0, 6PB1 [10] | 6PB0 (site-2), 6PB1 (site-1, site-2) |
| Glucose-dependent insulinotropic polypeptide receptor (GIPR) | 7DTY [11] | 7DTY (site-2, site-3) |
| Growth hormone releasing hormone receptor (GHRHR) | 7CZ5 [12] | N/A |
| Vasoactive intestinal polypeptide receptor 1 (VIP1R) | 6VN7 [13] | 6VN7 (site-2, site-3) |

**Supplementary table references**

[1] Jennings, L.J., Xu, Q.W., Firth, T.A., Nelson, M.T., Mawe, G.M., 1999. Cholesterol inhibits spontaneous action potentials and calcium currents in guinea pig gallbladder smooth muscle. Am J Physiol 277(5):G1017-1026.

[2] Buenaventura, T., Bitsi, S., Laughlin, W.E., Burgoyne, T., Lyu, Z., Oqua, A.I., et al., 2019. Agonist-induced membrane nanodomain clustering drives GLP-1 receptor responses in pancreatic beta cells. PLoS Biol 17(8):e3000097.

[3] Lucey, M., Ashik, T., Marzook, A., Wang, Y., Goulding, J., Oishi, A., et al., 2021. <strong>Acylation of the incretin peptide exendin-4 directly impacts GLP-1 receptor signalling and trafficking</strong>. Molecular pharmacology:MOLPHARM-AR-2021-000270.

[4] Estall, J.L., Yusta, B., Drucker, D.J., 2004. Lipid raft-dependent glucagon-like peptide-2 receptor trafficking occurs independently of agonist-induced desensitization. Mol Biol Cell 15(8):3673-3687.

[5] Emery, A.C., Liu, X.H., Xu, W., Eiden, M.V., Eiden, L.E., 2015. Cyclic Adenosine 3',5'-Monophosphate Elevation and Biological Signaling through a Secretin Family Gs-Coupled G Protein-Coupled Receptor Are Restricted to a Single Adenylate Cyclase Isoform. Mol Pharmacol 87(6):928-935.

[6] Tovey, S.C., Taylor, C.W., 2013. Cyclic AMP directs inositol (1,4,5)-trisphosphate-evoked Ca2+ signalling to different intracellular Ca2+ stores. J Cell Sci 126(Pt 10):2305-2313.

[7] Mahavadi, S., Bhattacharya, S., Kim, J., Fayed, S., Al-Shboul, O., Grider, J.R., et al., 2013. Caveolae-dependent internalization and homologous desensitization of VIP/PACAP receptor, VPAC₂, in gastrointestinal smooth muscle. Peptides 43:137-145.

[8] Zhao, L.H., Ma, S., Sutkeviciute, I., Shen, D.D., Zhou, X.E., de Waal, P.W., et al., 2019. Structure and dynamics of the active human parathyroid hormone receptor-1. Science 364(6436):148-153.

[9] Cong, Z., Chen, L.-N., Ma, H., Zhou, Q., Zou, X., Ye, C., et al., 2021. Molecular insights into ago-allosteric modulation of the human glucagon-like peptide-1 receptor. Nature communications 12(1):3763.

[10] Ma, S., Shen, Q., Zhao, L.-H., Mao, C., Zhou, X.E., Shen, D.-D., et al., 2020. Molecular Basis for Hormone Recognition and Activation of Corticotropin-Releasing Factor Receptors. Molecular cell 77(3):669-680.e664.

[11] Zhao, F., Zhang, C., Zhou, Q., Hang, K., Zou, X., Chen, Y., et al., 2021. Structural insights into hormone recognition by the human glucose-dependent insulinotropic polypeptide receptor. Elife 10.

[12] Zhou, F., Zhang, H., Cong, Z., Zhao, L.-H., Zhou, Q., Mao, C., et al., 2020. Structural basis for activation of the growth hormone-releasing hormone receptor. Nature communications 11(1):5205.

[13] Duan, J., Shen, D.-d., Zhou, X.E., Bi, P., Liu, Q.-f., Tan, Y.-x., et al., 2020. Cryo-EM structure of an activated VIP1 receptor-G protein complex revealed by a NanoBiT tethering strategy. Nature communications 11(1):4121.
