# Supplementary material for: Hepatocyte cholesterol content modulates glucagon receptor signalling
Source: Mol Metab. 2022 Jun 16;63:101530. doi: 10.1016/j.molmet.2022.101530 (PMC9254120; doi:10.1016/j.molmet.2022.101530)
Supplement: Multimedia component 3 [file mmc3.docx]

**Supplementary Methods**

**Hepatocyte cholesterol content modulates glucagon receptor signalling**

Emma Rose McGlone^1,2^, T. Bertie Ansell^3^, Cecilia Dunsterville^1^, Wanling Song^3,5^, David Carling^4^, Alejandra Tomas^1^, Stephen R Bloom^1^, Mark S. P. Sansom^3,*^, Tricia Tan^1^, Ben Jones^1,*^.

^1^ Department of Metabolism, Digestion and Reproduction, Imperial College London, London W12 0NN, United Kingdom.

^2^ Department of Surgery and Cancer, Imperial College London, London W12 0NN, United Kingdom.

^3^ Department of Biochemistry, University of Oxford, Oxford OX1 3QU, United Kingdom.

^4^ Cellular Stress Research Group, MRC London Institute of Medical Sciences, Imperial College London, London W12 0NN, United Kingdom.

^5^ Current address: Rahko, Clifton House, 46 Clifton Terrace, Finsbury Park, London N4 3JP, United Kingdom.

* Corresponding authors

**Supplementary methods**

## cAMP accumulation

Huh7-GCGR cells plated in 96-well plates, or primary mouse or human hepatocytes in suspension, were stimulated with agonist in serum-free medium for 10 min at 37°C. The phosphodiesterase inhibitor 3-isobutyl-1-methylxanthine (IBMX; 100 µM) was included in primary hepatocyte assays only. Where indicated, results were normalised to the 10 µM forskolin response. Where indicated, 10 ng/ml pertussis toxin (Sigma-Aldrich) was applied overnight in advance of the assay to inhibit Gα_i_. Cells were lysed, and cAMP was assayed by immunoassay (Cisbio HTRF cAMP Dynamic 2).

## cAMP detection by live cell imaging

Huh7-GCGR cells were seeded into 96-well black clear-bottomed plates and transduced with cADDis biosensors [1] (Montana Molecular) in a BacMam vector according to the manufacturer’s instructions. Specifically, sensors for total cytosolic cAMP (Green Up cADDis), lipid raft membrane cAMP (Green Membrane-Targeted FMP cADDis) and non-raft membrane cAMP (Red Membrane-Targeted fS15 cADDis) were used, with the latter two co-transduced to be used in the same imaging experiment. After 24 hours, cells were incubated with cholesterol-modulating treatments for 30 min and then washed prior to the assay. cAMP responses were monitored in real time by epifluorescence using an automated Nikon Ti2 widefield microscope with 10⨉ or 20⨉ air objectives. Single wavelength recordings were made using a Hammamatsu ORCA-Flash 4.0 LT+ camera, and dual wavelength recordings were made using a Photometrics Iris 15 camera after passing through an emission image splitter (OptoSplit III, Cairn Research). Image analysis was performed using Fiji v1.53q and included flat-field illumination correction using BaSiC [2], drift correction by SIFT and segmentation of cells expressing the sensor according to signal intensity. Responses were expressed first as treatment-induced fractional change from baseline, followed by normalisation to the peak response to 100 µM IBMX + 10 µM forskolin to saturate the sensor. Results confirmed that glucagon-induced responses fell within the sensor dynamic range.

## PKA activation assay

Huh7-GCGR cells in 96-well black clear-bottomed plates were reverse-transfected with the protein kinase A (PKA) FRET biosensor pcDNA3-AKAR4-NES [3] (a gift from Jin Zhang, Addgene plasmid #64727) using Lipofectamine 2000. After 24 hours, cells were incubated with cholesterol-modulating treatments for 30 min and then washed prior to the assay. PKA responses were monitored in real time by FRET microscopy with illumination at 435 nm, with dual wavelength recordings made using an image splitter as in Section 2.5 to simultaneously record CFP and YFP emission channels. Images were recorded at baseline, 5 min post-agonist and 5 min post-IBMX/forskolin, with concentration responses obtained for the whole assay by sequentially moving between wells between time-points. Image analysis was performed similarly to as described in Section 1.2, with the YFP:CFP ratio from biosensor-expressing cell regions used to indicate PKA activation. Glucagon-induced responses were expressed as a percentage of the peak response to 100 µM IBMX + 10 µM forskolin to saturate the sensor, followed by subtraction of the vehicle response.

## Fluorescent glucagon uptake assay

Huh7-GCGR cells in 96-well black, clear bottom plates were stimulated with FITC-GCG for 10 minutes at 37°C in serum-free medium. Unbound agonist was removed by washing three times with HBSS, followed by fixation with 2% paraformaldehyde (PFA) for 10 minutes at room temperature. High content imaging was performed by epifluorescence at 20⨉, with 9 fields-of-view acquired per well, and internalised agonist was quantified from epifluorescence images as previously described [4].

## Mini-G_s_ NanoBRET assay

Huh7-GCGR cells were seeded into white 96-well plates and co-transfected using Lipofectamine 2000 with mini-G_s_ tagged with nanoluciferase [5] (a gift from Prof Nevin Lambert, Medical College of Georgia) and the membrane marker KRAS-venus, as previously described [6]. At 36 hours, cells were pre-loaded with cholesterol for 30 min. After washing, cells were treated with 100 nM glucagon or vehicle (HBSS) for 30 min before addition of Furimazine (1:50 dilution), and luminescence signal was recorded using a Flexstation 3 plate reader. BRET signal was expressed as the ratio of venus emission (535 nm) to nanoluciferase emission (460 nm).

## Coarse-grained molecular dynamics simulations and analysis

Structures of the GCGR in inactive and active conformations were derived from the Protein Data Bank (PDB ID: inactive 5XEZ, active 6LMK) [7; 8]. Additional components (including bound glucagon in 6LMK) were removed and missing loops and/or incomplete residues were modelled using Modeller [9; 10]. The GCGR was coarse-grained using martinize.py [11] and embedded in an asymmetric lipid bilayer membrane comprising POPC (20%), DOPC (20%), POPE (5%), DOPE (5%), sphingomyelin (15%), GM3 (10%) and cholesterol (25%) in the upper leaflet and POPC (5%), DOPC (5%), POPE (20%), DOPE (20%), POPS (8%), DOPS (7%), PIP_2_ (10%) and cholesterol (25%) in the lower leaflet using insane.py [12]. The membrane composition was selected to recapitulate the main components of plasma membranes, as identified from lipodomics data [13; 14]. The recently re-paramiterised MARTINI 3 forcefield [15] may be able to more readily recapitulate realistic membrane behaviour however, MARTINI 3 parameters for cholesterol are still under development. Thus, the MARTINI 2.2 coarse grained (CG) forcefield [16] was used to describe all components and cholesterol was modelled with the virtual site descriptor [17]. The ElNeDyn elastic network was applied to each protein with a cut-off of 0.9 nm and a spring force constant of 500 kJ mol^-1^ nm^-2^ [18]. Each system was solvated using MARTINI water [16] and ~0.15 M NaCl before independent minimization and equilibration steps. Simulations were run identically to those described previously [10] for 10x 10 μs of each GCGR conformation. The linear constraint parameters, lincs_iter=1 and lincs_order=4 were used however recent work suggests higher values (e.g. lincs_iter=2 and lincs_order=12) may be necessary to avoid artefactual temperature gradients within bilayers containing lipids with virtual sites [19]. The GROMACS 5.14 and 2019 simulation packages were used to perform simulations (www.gromacs.org). Cholesterol interactions with GCGR were analysed using PyLipID [20] with a 0.55/0.8 nm double cut-off to define lipid contact. Binding sites and kinetic analysis (i.e. derived residence times) were calculated by PyLipID as described in [20].

## Atomistic molecular dynamics simulation

The top ranked cholesterol binding pose at site-4 (as identified from the coarse-grained trajectories of the inactive GCGR conformation using PyLipID [20]), was backmapped to atomistic resolution using CG2AT [21]. In the top ranked pose the cholesterol β3-hydroxy group is positioned approximately at the bilayer midplane. The protein conformation was backmapped to that of the inactive GCGR crystal structure [7]. The GCGR-cholesterol complex was embedded in a simplified POPC bilayer with a reduced system size of 7 x 7 x 11 nm3 using the CHARMM-GUI bilayer builder [22]. The system was solvated with ~0.15 M NaCl and TIP3P water [23]. Three replicates were independently minimized and equilibrated in 2 x 5 ns NVT and NPT steps with position restraints applied to the protein backbone and cholesterol heavy atoms. Each replicate was simulated for 1 μs (3 μs total) using the GROMACS 2019 simulation software (www.gromacs.org). The CHARMM-36 forcefield was used for all components [24]. The temperature was maintained at 310 K using the Nosé-Hoover thermostat (𝜏_t_=0.5 ps) [25] and the pressure was maintained at 1 bar using the Parrinello-Rahman barostat (𝜏_p_=2.0 ps, compressibility=4.5x 10^-5^ bar^-1^) [26]. The timestep was 2 fs. A dispersion correction was not applied. Lennard-Jones interactions were smoothly switched between 1.0 and 1.2 nm using the force-switch modifier. Electrostatic interactions were modelled using the Particle-Mesh-Ewald (PME) model [27]. LINCS constraints [28] were applied to maintain the equilibrium lengths of H-atom bonds. Control replicates (3x 500 ns) were run identically to described above with a cholesterol pose at site-4 which was inverted by 180° such that the β_3_-hydroxyl group was in proximity to the lipid phosphate groups.

## Computational binding saturation curves

Binding saturation curves and site affinities were calculated in accordance with a recently published method [29]. GCGR (PDB ID: active 6LMK) was simulated in binary POPC:cholesterol bilayers of increasing cholesterol content (1%, 2.5%, 5%, 10%, 15%, 30%, 40%). Each system was simulated for 5x 5 μs with simulation parameters as described above. The concentration of free cholesterol (%) was defined as the mean number of unbound cholesterol (> 0.8 nm from the GCGR transmembrane domain) divided by the total number of lipids in the bilayer. Site occupancies correspond to the mean occupancy of 6 residues at each site, as obtained by PyLipID. Apparent dissociation constants (K_d_^app^s) were calculated via fitting to equation 1 using Prism 9.2.0 for MacOS (Graphpad).

$$\left[ 1 \right] Occupancy= \frac{B_{max}^{app}+{[CHOL]}_{free}}{K_{d}^{app}+ {[CHOL]}_{free}}$$

**Supplementary methods references**

[1] Tewson, P.H., Martinka, S., Shaner, N.C., Hughes, T.E., Quinn, A.M., 2016. New DAG and cAMP Sensors Optimized for Live-Cell Assays in Automated Laboratories. J Biomol Screen 21(3):298-305.

[2] Peng, T., Thorn, K., Schroeder, T., Wang, L., Theis, F.J., Marr, C., et al., 2017. A BaSiC tool for background and shading correction of optical microscopy images. Nature communications 8(1):14836.

[3] Herbst, K.J., Allen, M.D., Zhang, J., 2011. Spatiotemporally regulated protein kinase A activity is a critical regulator of growth factor-stimulated extracellular signal-regulated kinase signaling in PC12 cells. Mol Cell Biol 31(19):4063-4075.

[4] Pickford, P., Lucey, M., Rujan, R.M., McGlone, E.R., Bitsi, S., Ashford, F.B., et al., 2021. Partial agonism improves the anti-hyperglycaemic efficacy of an oxyntomodulin-derived GLP-1R/GCGR co-agonist. Molecular metabolism 51:101242.

[5] Wan, Q., Okashah, N., Inoue, A., Nehmé, R., Carpenter, B., Tate, C.G., et al., 2018. Mini G protein probes for active G protein-coupled receptors (GPCRs) in live cells. The Journal of biological chemistry 293(19):7466-7473.

[6] Marzook, A., Chen, S., Pickford, P., Lucey, M., Wang, Y., Corrêa, I.R., Jr., et al., 2021. Evaluation of efficacy- versus affinity-driven agonism with biased GLP-1R ligands P5 and exendin-F1. Biochem Pharmacol 190:114656.

[7] Zhang, H., Qiao, A., Yang, D., Yang, L., Dai, A., de Graaf, C., et al., 2017. Structure of the full-length glucagon class B G-protein-coupled receptor. Nature 7:339.

[8] Qiao, A., Han, S., Li, X., Li, Z., Zhao, P., Dai, A., et al., 2020. Structural basis of Gs and Gi recognition by the human glucagon receptor. Science 367(6484):1346-1352.

[9] Fiser, A., Sali, A., 2003. Modeller: generation and refinement of homology-based protein structure models. Methods Enzymol 374:461-491.

[10] Ansell, T.B., Song, W., Sansom, M.S.P., 2020. The Glycosphingolipid GM3 Modulates Conformational Dynamics of the Glucagon Receptor. Biophys J 119(2):300-313.

[11] de Jong, D.H., Singh, G., Bennett, W.F., Arnarez, C., Wassenaar, T.A., Schafer, L.V., et al., 2013. Improved Parameters for the Martini Coarse-Grained Protein Force Field. J Chem Theory Comput 9(1):687-697.

[12] Wassenaar, T.A., Ingolfsson, H.I., Bockmann, R.A., Tieleman, D.P., Marrink, S.J., 2015. Computational Lipidomics with insane: A Versatile Tool for Generating Custom Membranes for Molecular Simulations. J Chem Theory Comput 11(5):2144-2155.

[13] Lorent, J.H., Levental, K.R., Ganesan, L., Rivera-Longsworth, G., Sezgin, E., Doktorova, M., et al., 2020. Plasma membranes are asymmetric in lipid unsaturation, packing and protein shape. Nat Chem Biol 16(6):644-652.

[14] Sampaio, J.L., Gerl, M.J., Klose, C., Ejsing, C.S., Beug, H., Simons, K., et al., 2011. Membrane lipidome of an epithelial cell line. Proc Natl Acad Sci U S A 108(5):1903-1907.

[15] Souza, P.C.T., Alessandri, R., Barnoud, J., Thallmair, S., Faustino, I., Grunewald, F., et al., 2021. Martini 3: a general purpose force field for coarse-grained molecular dynamics. Nat Methods 18(4):382-388.

[16] Marrink, S.J., Risselada, H.J., Yefimov, S., Tieleman, D.P., de Vries, A.H., 2007. The MARTINI force field: coarse grained model for biomolecular simulations. J Phys Chem B 111(27):7812-7824.

[17] Melo, M.N., Ingolfsson, H.I., Marrink, S.J., 2015. Parameters for Martini sterols and hopanoids based on a virtual-site description. J Chem Phys 143(24):243152.

[18] Periole, X., Cavalli, M., Marrink, S.J., Ceruso, M.A., 2009. Combining an Elastic Network With a Coarse-Grained Molecular Force Field: Structure, Dynamics, and Intermolecular Recognition. J Chem Theory Comput 5(9):2531-2543.

[19] Thallmair, S., Javanainen, M., Fabian, B., Martinez-Seara, H., Marrink, S.J., 2021. Nonconverged Constraints Cause Artificial Temperature Gradients in Lipid Bilayer Simulations. J Phys Chem B 125(33):9537-9546.

[20] Song, W., Corey, R.A., Ansell, T.B., Cassidy, C.K., Horrell, M.R., Duncan, A.L., et al., 2022. PyLipID: A Python package for analysis of protein-lipid interactions from MD simulations. J Chem Theory Comput 18(2):1188–1201.

[21] Vickery, O.N., Stansfeld, P.J., 2021. CG2AT2: an Enhanced Fragment-Based Approach for Serial Multi-scale Molecular Dynamics Simulations. J Chem Theory Comput 17(10):6472-6482.

[22] Lee, J., Patel, D.S., Stahle, J., Park, S.J., Kern, N.R., Kim, S., et al., 2019. CHARMM-GUI Membrane Builder for Complex Biological Membrane Simulations with Glycolipids and Lipoglycans. J Chem Theory Comput 15(1):775-786.

[23] Jorgensen, W.L., Chandrasekhar, J., Madura, J.D., Impey, R.W., Klein, M.L., 1983. Comparison of simple potential functions for simulating liquid water. The Journal of Chemical Physics 79(2):926-935.

[24] Huang, J., MacKerell, A.D., Jr., 2013. CHARMM36 all-atom additive protein force field: validation based on comparison to NMR data. J Comput Chem 34(25):2135-2145.

[25] Nosé, S., 1984. A molecular dynamics method for simulations in the canonical ensemble. Molecular Physics 52(2):255-268.

[26] Parrinello, M., Rahman, A., 1981. Polymorphic transitions in single crystals: A new molecular dynamics method. Journal of Applied Physics 52(12):7182-7190.

[27] Darden, T., York, D., Pedersen, L., 1993. Particle mesh Ewald: An N⋅log(N) method for Ewald sums in large systems. The Journal of Chemical Physics 98(12):10089-10092.

[28] Hess, B., Bekker, H., Berendsen, H.J.C., Fraaije, J.G.E.M., 1998. LINCS: A linear constraint solver for molecular simulations. J Comput Chem 18(12):1463-1472.

[29] Ansell, T.B., Curran, L., Horrell, M.R., Pipatpolkai, T., Letham, S.C., Song, W., et al., 2021. Relative Affinities of Protein-Cholesterol Interactions from Equilibrium Molecular Dynamics Simulations. J Chem Theory Comput.
